# Long Non-Coding RNA THOR Depletion Inhibits Human Non-Small Cell Lung Cancer Cell Growth

**DOI:** 10.3389/fonc.2021.756148

**Published:** 2021-11-17

**Authors:** Peng-Fei Jiao, Pei-jun Tang, Dan Chu, Ya-meng Li, Wei-hua Xu, Gao-Fei Ren

**Affiliations:** ^1^ Department of Respiration and Intensive, The First Affiliated Hospital of Zhengzhou University, Zhengzhou, China; ^2^ Department of Pulmonary, The Affiliated Infectious Diseases Hospital of Soochow University, The Fifth People’s Hospital of Suzhou, Suzhou, China; ^3^ Department of Cardiothoracic Surgery, the Second Affiliated Hospital of Soochow University, Suzhou, China

**Keywords:** NSCLC, Lnc-THOR, IGF2BP1, cell growth, signaling

## Abstract

Long non-coding RNA (LncRNA) THOR (Lnc-THOR) is expressed in testis and multiple human malignancies. Lnc-THOR association with IGF2BP1 (IGF2 mRNA-binding protein 1) is essential for stabilization and transcription of IGF2BP1 targeted mRNAs. We tested its expression and potential functions in non-small cell lung cancer (NSCLC). In primary NSCLC cells and established cell lines, Lnc-THOR shRNA or CRISPR/Cas9-mediated knockout (KO) downregulated IGF2BP1 target mRNAs (*IGF2*, *Gli1*, *Myc* and *SOX9*), inhibiting cell viability, growth, proliferation, migration and invasion. Significant apoptosis activation was detected in Lnc-THOR-silenced/-KO NSCLC cells. Conversely, ectopic overexpression of Lnc-THOR upregulated IGF2BP1 mRNA targets and enhanced NSCLC cell proliferation, migration and invasion. RNA-immunoprecipitation and RNA pull-down assay results confirmed the direct binding between Lnc-THOR and IGF2BP1 protein in NSCLC cells. Lnc-THOR silencing and overexpression were ineffective in IGF2BP1-KO NSCLC cells. Forced IGF2BP1 overexpression failed to rescue Lnc-THOR-KO NSCLC cells. *In vivo*, intratumoral injection of Lnc-THOR shRNA adeno-associated virus potently inhibited A549 xenograft tumor growth in nude mice. At last we show that Lnc-THOR is overexpressed in multiple NSCLC tissues and established/primary NSCLC cells. Collectively, these results highlighted the ability of Lnc-THOR in promoting NSCLC cell growth by associating with IGF2BP1, suggesting that Lnc-THOR represents a promising therapeutic target of NSCLC.

## Introduction

Lung cancer is one major cause of human mortalities around the world (1, 2). Over 220,000 new cases and 140,000 deaths of lung cancer were reported in United States in 2019 alone (1, 2). Non-small cell lung cancer (NSCLC), including adenocarcinoma (ADC), squamous cell carcinoma (SCC), large cell carcinoma (LCC) and adenosquamous cell carcinoma (ASC), accounts for over 80-85% of all lung cancer (1, 2). Significant achievements have been made in clinical treatments of NSCLC, including radiotherapy, chemotherapy, surgery, and recently-developed molecular targeted agents (3–5). Yet the five-year overall survival for NSCLC patients remains at approximately 15-20% (3–5). The prognosis of this devastating disease is even worse in certain countries (1, 2, 6, 7). Therefore, it is urgent to further explore the underlying pathological mechanisms required for NSCLC tumorigenesis and progression (3, 4).

Long non-coding RNAs (LncRNAs) are a large family of conserved single strand RNA molecules over 200-nt long (8–10). LncRNAs could be able to alter expression and/or functions of genes through acting as microRNA (miRNA) spongers or binding to RNA binding proteins (RBPs) (8–10). Dysregulation of LncRNAs is commonly detected in NSCLC (11, 12), which is implicated in tumorigenesis and cancer progression (11, 12). LncRNAs are involved in the epigenetic regulation of genes essential for NSCLC growth (13–19). Nie and colleagues demonstrated that LncRNA UCA1 is upregulated in NSCLC, predicting poor survival time, and is an independent risk factor of prognosis (19). LncRNA UCA1 increased ERBB4 expression *via* sponging miR-193a, thereby promoting NSCLC cell growth (19). Another LncRNA PVT1 expression is elevated in NSCLC, correlating with histological grade, lymph node metastasis and poor overall survival (20). LncRNA PVT1 silencing potently inhibited NSCLC cell proliferation, migration, and invasion (20).

Insulin-like growth factor 2 (IGF2) mRNA-binding protein 1 (IGF2BP1) belongs to the IGF2BP RNA-binding family proteins (21), required for stabilization and translation of several mRNAs in human cancer (22, 23), including *Gli1* (*glioma-associated oncogene homolog 1*), *Myc*, *IGF2* and *SOX9* (24). Recent studies have discovered a conserved IGF2BP1-binding LncRNA, namely THOR (Lnc-THOR) (24). It is expressed in testis and multiple human cancers (24). Lnc-THOR binds to IGF2BP1 (24), essential for the stabilization and transcriptional activation of IGF2BP1-dependent mRNAs (24, 25). Lnc-THOR depletion could result in significant anti-cancer cell activity (24–31). We here tested the expression and potential functions of Lnc-THOR in NSCLC.

## Materials and Methods

### Ethics

All the methods applied in this study were carried out according to the ethics guidelines of Zhengzhou University.

### Chemicals, Reagents and Antibodies

The cleaved caspase antibody sampler kit (#9929), IGF2BP1, (#8482) and β-Tubulin (#2146) were purchased from Cell Signaling Technologies (Beverly, MA). Cell culture reagents were obtained from Hyclone Co. (Logan, UT). Puromycin, polybrene and other chemicals were purchased from Sigma-Aldrich (St. Louis, Mo).

### Cell Culture

Established NSCLC cell lines, A549 and H1299, were from Dr. Li at Wenzhou Medical University (32). Cells were grown in RPMI-1640 medium containing 10% FBS. The primary human NSCLC cells, derived from three different patients, “pCan-1”, “pCan-2” and “pCan-3”, as well as the primary human lung epithelial cells (“pEpi”), were from Dr. Shi at The Second Affiliated Hospital of Soochow University, and were cultured as described (33). The written-informed consent was obtained from each participant. The protocols of using human cells were approved by the Ethics Committee of Zhengzhou University, in according to the principles of Declaration of Helsinki.

### Patients and Tissue Samples

A set of 10 pairs of NSCLC tumor tissues and the corresponding adjacent normal lung tissues (over two cm away from the boundary of tumor tissue) were obtained from primary NSCLC patients with tumor resection. The patients were administrated at the First affiliated Hospital of Zhengzhou University and received no preoperative treatments. Human testis tissues were from a patient with post-traumatic orchiectomy. The written informed consent was obtained each patient. Human tissues were stored in liquid nitrogen immediately after resection. The protocols of using human tissues were approved by the Ethics Committee of Zhengzhou University, in according to the principles of Declaration of Helsinki.

### Quantitative Real Time-PCR (qRT-PCR)

In brief, total RNA was extracted from cultured cells and tissues by the TRIzol reagents (Invitrogen, Carlsbad, CA). The retrieved RNA was reversely transcribed into cDNA by an SuperScript™ II Reverse Transcriptase Kit (Invitrogen). qRT-PCR was performed by using SYBR Premix Ex Taq™ (Takara, Shanghai, China) under the ABI Prism 7900 Fast Real-Time PCR system (Applied bioscience, Shanghai, China). *GAPDH* was tested as the reference gene, with 2^−ΔΔCt^ method applied for data quantification. The mRNA primers were from Dr. Pan at Shanghai Jiao Tong University (34).

### Western Blotting

Equal amounts of total protein lysates (40 μg per lane) were resolved by sodium dodecyl sulfate–polyacrylamide gel (SDS-PAGE) electrophoresis and transferred to polyvinylidene difluoride (PVDF) blots. The blots were blocked and immuno-blotted with the primary antibodies overnight, following by incubation with the corresponding secondary antibodies. An enhanced chemiluminescence (ECL) detection kit (Amersham, Buckinghamshire, UK) was applied to detect targeted protein bands. The ImageJ software from NIH was utilized for data quantification.

### Lnc-THOR shRNA

The lentiviral particles, encoding shRNAs against non-overlapping sequence of Lnc-THOR (“sh-S1/sh-S2”), were provided by Dr. Pan at Shanghai Jiao Tong University (34). NSCLC cells were plated at a density of 1 ×10 ^5^ cells/well into six-well plates (in polybrene containing complete medium) and were infected with the lentivirus (MOI=20). After 24h, puromycin (2.5 μg/mL) was added to select stable cells for 3-4 passages. Lnc-THOR knockdown in stable cells was verified by qRT-PCR assays. Control cells were infected lentiviral particles with scramble control shRNA (“shC”).

### Lnc-THOR Knockout (KO)

NSCLC cells were seeded into six-well plates (at 1.0 × 10^5^ cells per well) and were transfected with a LentiCas9-puro construct (Genechem). Stable Cas9 NSCLC cells were established after puromycin selection. Cells were then transfected with a pSpCas9(BB)-2A (PX458) plasmid encoding sgRNA against Lnc-THOR [provided by Dr. Pan at Shanghai Jiao Tong University (34)]. The transfected cells were distributed into 96-well plates and subject to Lnc-THOR KO screening. The single stable Lnc-THOR KO NSCLC cells were then established. Control cells were transduced with the Cas9 control empty vector (“Cas9-C”).

### Lnc-THOR Overexpression

The GV248 lentiviral construct encoding the full-length Lnc-THOR was provided again by Dr. Pan (34), that was transfected to primary NSCLC cells. Cells were subject to puromycin (2.5 μg/mL) selection for another 4-5 passages. Two stable lines of NSCLC cells with Lnc-THOR-expressing construct, “OE-L1” and “OE-L2”, were established. Lnc-THOR overexpression in stable cells was verified by qRT-PCR assay. Control cells were infected with the empty vector (“Vec”).

### Cell Viability

NSCLC cells were plated (4 × 10 ^3^ cells per well) in 96-well microplates and cultured for 96h. Afterwards, 10 μL per well of CCK-8 solution was added for 2h. CCK-8 optical density (OD) was measured at 490 nm.

### Colony Formation

NSCLC cells were grown in a 10-cm culturing dish at (2 × 10^4^ cells per dish). Medium was changed every two days for a total of 12 days. Afterwards, the cell colonies were fixed with methanol, washed with PBS and stained with Giemsa. Finally, the number of colonies (with ≥50 cells per colony) were counted.

### EdU Staining

The 5-ethynyl-20-deoxyuridine (EdU) Apollo-488 Kit (Ribo-Bio, Guangzhou, China) was utilized. The detailed protocols were descried early (26). Briefly, NSCLC cells were seeded into 12-well plates at 0.5 × 10^5^ cells per well and were cultured for 96h. Cell nuclei were then stained with EdU (10 μM) and DAPI, visualized under a fluorescent microscope (Leica, Beijing, China).

### Cell Migration and Invasion Assays

The *in vitro* cell migration was tested by using 24-well “Transwell” chambers (Becton Dickinson, Shanghai, China). In brief, NSCLC cells, at 3 × 10^4^ per well, were seeded in the upper surface of the Transwell chamber in basic DMEM. The lower chamber was filled with complete medium (with 10% FBS) to attract cells. After 24h, non-migrated NSCLC cells were removed carefully from the top well with a cotton swab, with NSCLC cells on the bottom fixed and stained. The migrated cells were photographed (Olympus, Tokyo, Japan). For *in vitro* invasion assays, the “Transwell” chambers were always coated with Matrigel (Sigma). Data quantification was reported early (26).

### Caspase-3 Activity Assay

Briefly, NSCLC cells were grown in 12-well plates for 72h. Cells were then harvested and tested for caspase-3 activity by a colorimetric assay kit (BioVision, Mountain View, CA) according to the attached protocol.

### ssDNA ELISA

NSCLC cells were grown in 12-well plates at 0.5 × 10 ^5^ cells per well for 72h. Cells were then rinsed with cold PBS, fixed with ice-cold methanol, and incubated with 100% formamide (Roth, Karlsruhe, Germany). Afterwards, cells were incubated with 3% H_2_O_2_ and blocked by non-fat dry milk. A ssDNA ELISA Kit (Millipore, Billerica, MA) was then utilized for detection total ssDNA contents based on the attached protocols. ssDNA absorbance in each well was detected at 405 nm.

### TUNEL Assay

Briefly, NSCLC cells were grown into 12-well plates for 96h. Cells were fixed with 4% formaldehyde, followed by permeabilization as described (35). Cells were then incubated with TUNEL reaction mixture containing nucleotide mixture and terminal deoxynucleotidyl transferase (TdT), co-stained with DAPI, washed with PBS, and detected under a fluorescence microscope.

### JC-1 Mitochondrial Membrane Potential (ΔΨ_m_) Assay

Mitochondrial membrane potential (ΔΨ_m_) reduction, or mitochondrial depolarization, was detected by JC-1 staining. NSCLC cells were grown in 12-well plates for 72h and stained JC-1 (5 μg/mL) for 30 min at 37°C. JC-1 green monomer fluorescence intensity (at 490 nm) was detected using a fluorescence spectrofluorometer (Titertek Fluoroscan II; Flow Laboratories, North Ryde, Australia). JC-1 images, intergrading both green (at 490 nm) and red (at 625 nm) fluorescence channels, were presented.

### RNA-Immunoprecipitation (RIP)

The pCan-1 primary NSCLC cells and A549 cells were incubated with 0.3% formaldehyde and glycine (0.125 M), and cell pellets re-suspended in RIP buffer described early (36). An anti-IGF2BP1 antibody (#8482, Cell Signaling Tech, Beverly, MA) was added to the cell lysates, and IGF2BP1-bound pellets were washed, re-suspended and the magnetic beads were added. The mixture was incubated on a rotator at 4°C overnight. After collecting the magnetic bead-bound complex, the proteinase K was utilized. qRT-PCRs assay were then performed to examine IGF2BP1-bound RNA.

### RNA Pull-Down

Biotin-labeled full-length Lnc-THOR was provided by Dr. Chen’s Lab at Jiangsu University (26), and was dissolved in RNA structure buffer (Beyotime, Wuxi, China) to obtain an appropriate secondary structure. For RNA pull-down assay, 600 μg nuclei lysates of the pCan-1 primary NSCLC cells and A549 cells were mixed with folded Biotin-Lnc-THOR and Dynabeads MyOne Streptavidin C1 magnetic beads [“Beads”, provided by Dr. Chen (26)]. The mixture was incubated on a rotator at 4°C overnight. Beads were washed three times. The bound proteins were eluted in 60 μL protein lysis buffer, separated by the SDS-PAGE, and examined by Western blotting assays.

### IGF2BP1 KO

NSCLC cells were seeded into six-well plates (at 1.0 × 10^5^ cells per well) and were transfected with a LentiCas9-puro construct (Genechem). Stable Cas9 NSCLC cells were established after puromycin selection. Cells were then transfected with the CRISPR/Cas9-IGF2BP1-KO construct [from Dr. Cheng’s group at Soochow University (37)] and were then distributed to 96-well plates, subject to IGF2BP1 KO screening. The IGF2BP1-KO monoclonal stable cells were then established, with IGF2BP1 expression examined by Western blotting and qRT-PCR assays.

### IGF2BP1 Overexpression

The recombinant adenovirus encoding IGF2BP1-expressing pSUPER-puro construct was from Dr. Zhao at Soochow University (38). NSCLC cells were grown in six-well tissue culture plates (at 0.6 × 10^5^ cells per well) and were infected with the adenovirus for 48h. Stable cells were established by puromycin selection and IGF2BP1 overexpression verified by Western blotting and qRT-PCR assays.

### Xenograft Tumor Formation Assay

The nude mice, half male half female, aged 5-6 weeks, 18.5-19.0g in weights, were randomly divided into two groups, and were inoculated with A549 cells [at 6 ×10^6^ cells per mouse subcutaneously (s.c.)]. Nude mice were monitored every day, xenograft tumor weights and volumes were measured with a sliding caliper, and tumor volumes calculated using the formula (L×W^2^)/2. When the tumor volume was close to 100 mm^3^ (“Day-0”), mice were subject to intratumoral injection of Lnc-THOR shRNA (“sh-S1”) adeno-associated virus (AAV) or the scramble control shRNA (“shC”) AAV. All mice were sacrificed at the end of the experiments and the tumors were harvested. All animal studies were performed according to the standards of IACUC of Zhengzhou University, with the protocols approved by the Ethics Committee of Zhengzhou University.

### Statistical Analyses

All values were presented as mean ± standard deviation (SD). Statistical comparisons were performed by Student’s t-test (Excel 2007) between two groups or one-way ANOVA plus a Scheffe’ and Tukey Test (SPSS 23.0) for multiple comparisons. *P* < 0.05 was considered to indicate a significant difference. *In vitro* experiments were repeated at least three times, with similar results obtained.

## Results

### Lnc-THOR shRNA or KO Inhibits NSCLC Cell Viability, Proliferation, Migration and Invasion

**Figure 1:**
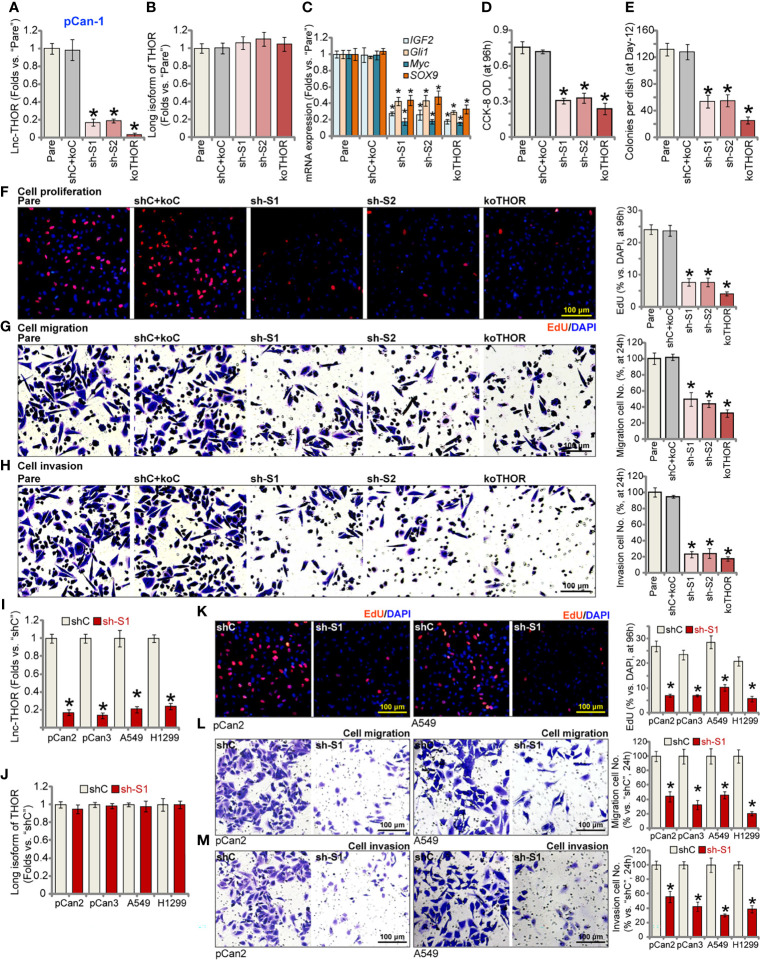
Lnc-THOR shRNA or KO inhibits NSCLC cell viability, proliferation, migration and invasion. Lnc-THOR shRNA (“sh-S1” or “sh-S2”, two different sequences)-expressing pCan-1 primary NSCLC cells, the CRISPR/Cas9-edited Lnc-THOR KO pCan-1 cells (“koTHOR”), or pCan-1 cells expressing the scramble control shRNA plus the Cas9-KO empty vector (“shC+koC”), were established; Expression of Lnc-THOR **(A)** and listed genes **(B, C)** was shown; Cells were cultured for applied time periods, and cell viability (CCK-OD, **D**), colony formation **(E)**, proliferation [EdU incorporation, **(F)**], migration and invasion [“Transwell” assays, **(G, H)**] were tested. Primary NSCLC cells (pCan-2 and pCan-3, derived from two different patients) or established cell lines (A549 and H1299), stably expressing the “sh-S1” Lnc-THOR shRNA or the scramble control shRNA (“shC”), were established; Expression of Lnc-THOR **(I)** and the long isoform of THOR **(J)** were shown; Cell proliferation **(K)**, migration **(L)** and invasion **(M)** were tested similarly. For the *in vitro* functional assays, the exact same number of viable cells of different genetic treatments were seeded onto each well/dish (“Day-0”/0h). “Pare” stands for the parental control cells. Data were presented as mean ± standard deviation (SD, n=5). *P < 0.05 *vs*. “Pare”/”shC” cells. The experiments were repeated five times, with similar results obtained. Scale Bar = 100 μm **(F–H, K–M)**.

The shRNA strategy was first employed to silence Lnc-THOR. As described, lentiviral particles encoding two different shRNA sequences, “sh-S1” and “sh-S2” [from Dr. Pan (34)], were transduced to pCan-1 primary NSCLC cells. Following selection *via* puromycin, stable cells were established. Alternatively, a Cas9-Lnc-THOR-KO construct [also from Dr. Pan (34)] was transfected to the Cas9-expressing pCan-1 cells. The transfected cells were subject to Lnc-THOR KO screening, and single stable cells established (“koTHOR” cells). Analyzing Lnc-THOR expression, *via* qRT-PCR assays, demonstrated that Lnc-THOR levels decreased over 80-90% in pCan-1 cells with the Lnc-THOR shRNA or the KO construct (**Figure 1A**). The linear THOR expression was however unchanged (**Figure 1B**). IGF2BP1 target mRNAs, including *IGF2*, *Gli1*, *Myc* and *SOX9* (25, 26, 34, 38), were robustly decreased in pCan-1 cells with Lnc-THOR shRNA or KO (**Figure 1C**).

Functional studies demonstrated that with Lnc-THOR silencing or KO, pCan-1 cell viability, or the CCK-8 OD, was significantly decreased (**Figure 1D**). Results in **Figure 1E** further showed that Lnc-THOR shRNA or KO potently inhibited pCan-1 cell colony formation. In addition, Lnc-THOR depletion robustly suppressed pCan-1 cell proliferation (**Figure 1F**), evidenced by decreased EdU-positive nuclei ratio (**Figure 1F**). In addition, Lnc-THOR silencing or KO largely inhibited pCan-1 cell *in vitro* migration and invasion, which were tested by “Transwell” (**Figure 1G**) and “Matrigel Transwell” (**Figure 1H**) assays, respectively. As expected, the scramble control shRNA plus the Cas9-KO empty vector (“shC+koC”) failed to significantly affect expression of Lnc-THOR and related genes (**Figures 1A–C**) and pCan-1 cell functions (**Figures 1D–H**).

The potential effect of Lnc-THOR in other NSCLC cells was studied next. Primary NSCLC cells derived from two other primary patients, pCan-2 and pCan-3, as well as the established cell lines (A549 and H1299), were tested. The lentiviral particles encoding Lnc-THOR shRNA (“sh-S1”) were added to the NSCLC cells. *Via* selection stable cells were established, showing dramatic Lnc-THOR silencing (**Figure 1I**). The long isoform of THOR expression was unchanged (**Figure 1J**). The nuclear EdU staining assay results, **Figure 1K**, showed that Lnc-THOR shRNA potently inhibited proliferation of the primary and established NSCLC cells. “Transwell” (**Figure 1L**) and “Matrigel Transwell” (**Figure 1M**) assay results further showed that Lnc-THOR silencing largely suppressed migration and invasion of the NSCLC cells.

### Lnc-THOR shRNA or KO Induces NSCLC Cell Apoptosis

**Figure 2:**
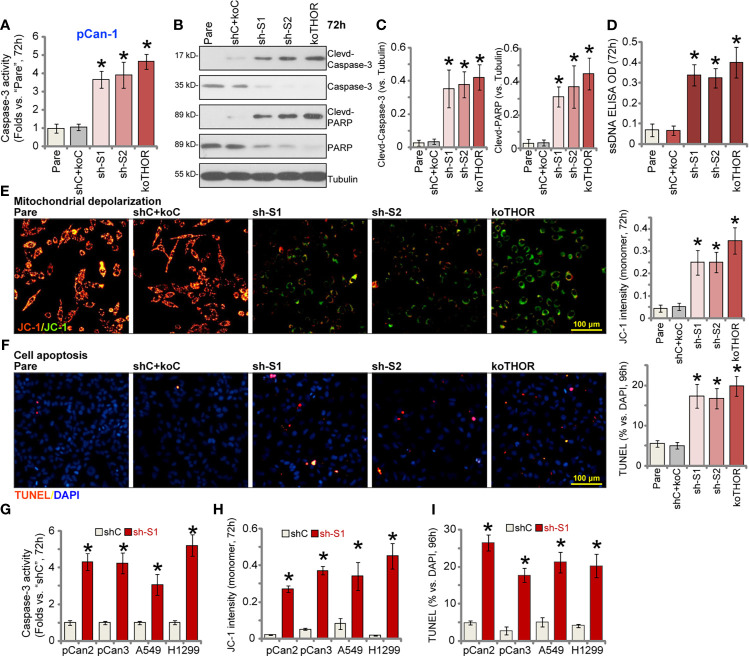
Lnc-THOR shRNA or KO induces NSCLC cell apoptosis. Lnc-THOR shRNA (“sh-S1” and “sh-S2”, two different sequences)-expressing pCan-1 cells, the CRISPR/Cas9-edited Lnc-THOR KO pCan-1 cells (“koTHOR”), or pCan-1 cells with the scramble control shRNA plus the Cas9-KO empty vector (“shC+koC”), were established and cultured for applied time periods; The relative caspase-3 activity **(A)**, expression of the apoptosis-associated proteins **(B, C)** and ssDNA contents [ELISA assays, **(D)**] were tested; Mitochondrial depolarization was tested by JC-1 green monomer accumulation **(E)**; Cell apoptosis was tested by nuclear TUNEL staining assay **(F)**. Primary NSCLC cells (pCan-2 and pCan-3, derived from two different patients) or established cell lines (A549 and H1299), stably expressing the “sh-S1” Lnc-THOR shRNA or the scramble control shRNA (“shC”), were established and cultured for applied time periods; The relative caspase-3 activity **(G)**, JC-1 green monomer intensity **(H)** and the TUNEL-positive nuclei ratio **(I)** were tested similarly. “Pare” stands for the parental control cells. Data were presented as mean ± standard deviation (SD, n=5). *P < 0.05 *vs*. “Pare”/”shC” cells. The experiments were repeated five times, with similar results obtained. Scale Bar = 100 μm **(D, E)**.

We next analyzed the potential effect of Lnc-THOR depletion on NSCLC cell apoptosis (26, 34). As shown, in stable pCan-1 cells expressing Lnc-THOR shRNA (“sh-S1” and “sh-S2”) or the Lnc-THOR-KO pCan-1 cells (“koTHOR”), the caspase-3 activity was significantly higher than that in the parental control cells (**Figure 2A**). Western blotting assay results showed that levels of cleaved caspase-3 and cleaved PARP [poly (ADP ribose) polymerase] were significantly increased in pCan-1 cells after Lnc-THOR silencing or KO (**Figures 2B, C**). Total caspase-3 and PARP levels were decreased (**Figure 2B**). In addition, ssDNA contents were dramatically increased (**Figure 2D**), indicating that Lnc-THOR depletion induced significant DNA break.

Further experimental results found that Lnc-THOR shRNA or KO induced mitochondrial depolarization in pCan-1 cells, evidenced by accumulation of JC-1 green monomer (**Figure 2E**). In Lnc-THOR-silenced or Lnc-THOR-KO pCan-1 cells, TUNEL-positive nuclei ratio was significantly increased, indicating apoptosis activation (**Figure 2F**). Therefore, Lnc-THOR depletion induced robust apoptosis activation in pCan-1 cells. The scramble control shRNA plus the Cas9-KO empty vector (“shC+koC”), unsurprisingly, failed to induce caspase-apoptosis activation in pCan-1 cells.

In pCan-2 and pCan-3 primary cells as well as in established cell lines (A549 and H1299), Lnc-THOR silencing by sh-S1 shRNA (see **Figure 1**) induced caspase-3 activation (**Figure 2G**) and mitochondrial depolarization (JC-1 green monomer intensity increase, **Figure 2H**). Significant apoptosis was detected as well in the Lnc-THOR-silenced NSCLC cells, as the TUNEL-positive nuclei ratio was significantly increased (**Figure 2I**). Together, these results showed that Lnc-THOR depletion induced NSCLC cell apoptosis.

### Lnc-THOR Overexpression Augments NSCLC Cell Growth and Motility

**Figure 3:**
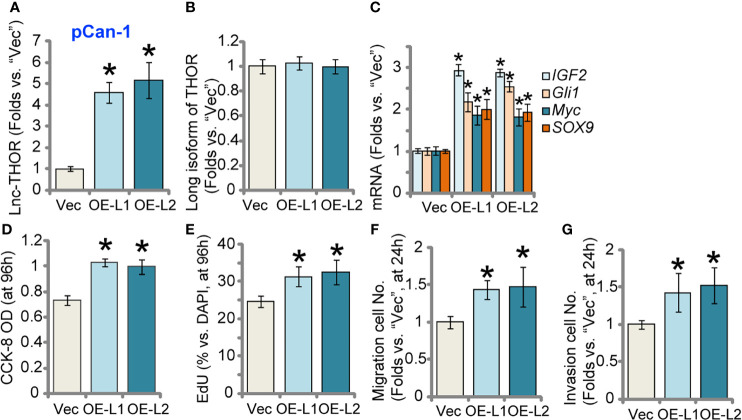
Lnc-THOR overexpression augments NSCLC cell growth and motility. pCan-1 cells, stably expressing the Lnc-THOR-expressing construct (“OE-L1 and OE-L2”, two stable cell lines) or the empty vector (“Vec”), were established; Expression of Lnc-THOR **(A)** and listed genes **(B, C)** was shown; Cells were cultured for applied time periods, cell viability [CCK-OD, **(D)**], proliferation [EdU incorporation, **(E)**], migration and invasion [“Transwell” assays, **(F)** and **(G)**] were tested, with results quantified. Data were presented as mean ± standard deviation (SD, n=5). *P < 0.05 *vs*. “Vec” cells. Experiments were repeated five times, with similar results obtained.

Next, a lentiviral construct encoding the full-length Lnc-THOR [from Dr. Pan (34)] was transduced to pCan-1 cells. Following selection by puromycin, two stable cell lines, “OE-L1” and “OE-L2”, were established. Examining Lnc-THOR expression, through qRT-PCR assays, confirmed that Lnc-THOR expression increased over 4-5 folds in OE-L1 cells and OE-L2 cells (**Figure 3A**), where the long isoform of THOR expression was unchanged (**Figure 3B**). IGF2BP1 target mRNAs, *IGF2*, *Gli1*, *Myc* and *SOX9*, were significantly increased after Lnc-THOR overexpression (**Figure 3C**). Functional studies demonstrated that Lnc-THOR overexpression augmented pCan-1 cell viability and proliferation, tested by CCK-8 OD (**Figure 3D**) and by recording the EdU-positive nuclei ratio (**Figure 3E**) assays, respectively. Moreover, *in vitro* migration (**Figure 3F**) and invasion (**Figure 3G**) were accelerated after Lnc-THOR overexpression. These results further supported a key role of Lnc-THOR in NSCLC cell progression.

### Lnc-THOR-Driven NSCLC Cell Growth Is Through Binding to IGF2BP1

**Figure 4:**
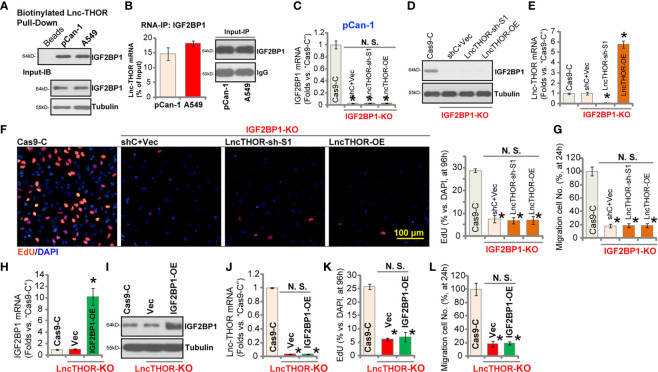
Lnc-THOR-driven NSCLC cell growth is through binding to IGF2BP1. Western blotting of the nuclear IGF2BP1 protein retrieved by biotin-labeled full-length Lnc-THOR in pCan-1 NSCLC cells and A549 cells **(A)**. qRT-PCR assay of Lnc-THOR enriched by IGF2BP1 protein in pCan-1 cells and A549 cells **(B)**. The stable pCan-1 cells expressing the CRISPR/Cas9-IGF2BP1-KO construct (“IGF2BP1-KO” cells) were further transduced with the “sh-S1” Lnc-THOR shRNA or the Lnc-THOR-expressing construct (“Lnc-THOR-OE”), control cells were transduced with the Cas9-KO empty vector (“Cas9-C”); Expression of *IGF2BP1* mRNA **(C)**, listed proteins **(D)** and Lnc-THOR **(E)** was shown; Cells were further cultured for applied time periods, cell proliferation and migration were tested by nuclear EdU staining **(F)** and “Transwell” **(G)** assays, respectively, with results quantified. The CRISPR/Cas9-edited Lnc-THOR KO pCan-1 cells (“koTHOR”) were further infected with recombinant adenovirus encoding the human IGF2BP1 expression construct (“OE-IGF2BP1”) or empty vector (“Vec”), control cells were with the Cas9-KO empty vector (“Cas9-C”); Expression of *IGF2BP1* mRNA **(H)**, listed proteins **(I)** and Lnc-THOR **(J)** was shown; Cells were further cultured for applied time periods, cell proliferation **(K)** and migration **(L)** were tested, with results quantified. Data were presented as mean ± standard deviation (SD, n=5). *P < 0.05 *vs.* “Cas9” cells. “N. S.” stands for non-statistical difference. The experiments were repeated five times, with similar results obtained. Scale Bar = 100 μm **(F)**.

Experiments were carried out to examine the possible association between Lnc-THOR and IGF2BP1 protein in NSCLC cells. Lnc-THOR pull-down assay results confirmed that IGF2BP1 protein in cell nuclei was precipitated with the biotinylated Lnc-THOR in pCan-1 primary NSCLC cells and A549 cells (**Figure 4A**). Additionally, by employing a RNA-Immunoprecipitation (RIP) assay, we further demonstrated the direct association between endogenous Lnc-THOR and IGF2BP1 protein in pCan-1 cells and A549 cells (**Figure 4B**). These results implied that Lnc-THOR directly associated with IGF2BP1 protein in NSCLC cells.

Whether Lnc-THOR-driven NSCLC cell growth was through binding to IGF2BP1 protein was tested next. Using the CRISPR/Cas9 gene-editing method [see (38)], we established the IGF2BP1-KO stable pCan-1 cells (IGF2BP1-KO). The qRT-PCR (**Figure 4C**) and Western blotting (**Figure 4D**) results confirmed IGF2BP1 KO in the stable cells, where Lnc-THOR expression was unchanged (**Figure 4E**). CRISPR/Cas9-induced IGF2BP1 KO potently inhibited pCan-1 cell proliferation (EdU staining assays, **Figure 4F**) and migration (**Figure 4G**). Importantly, altering Lnc-THOR expression (**Figure 4E**), by the Lnc-THOR shRNA (“sh-S1”, see **Figure 2**) or the Lnc-THOR-expressing construct (Lnc-THOR-OE, see **Figure 3**), failed to further affect cell proliferation (**Figure 4F**) and migration (**Figure 4G**) in IGF2BP1-KO cells. IGF2BP1 expression was not affected by Lnc-THOR shRNA or OE (**Figures 4C, D**). These results implied that Lnc-THOR-driven NSCLC cell growth was indeed through binding to IGF2BP1.

Next, whether ectopic IGF2BP1 overexpression could rescue the Lnc-THOR KO NSCLC cells was tested. IGF2BP1-expressing recombinant adenovirus, ad-IGF2BP1 [from Dr. Zhao (38)], was transduced to Lnc-THOR-KO pCan-1 cells, resulting in significant IGF2BP1 overexpression (**Figures 4H, I**). Ectopic IGF2BP1 overexpression, as expected, did not alter Lnc-THOR expression (**Figure 4J**). Functional studies demonstrated that Lnc-THOR KO-induced inhibition on cell proliferation (EdU-positive nuclei ratio reduction, **Figure 4K**) and migration (**Figure 4L**) were not alleviated by ectopic IGF2BP1 overexpression. Therefore, these results further supported that Lnc-THOR-IGF2BP1 association promoted NSCLC cell growth.

### Lnc-THOR shRNA Inhibits A549 Xenograft Tumor Growth in Nude Mice

**Figure 5:**
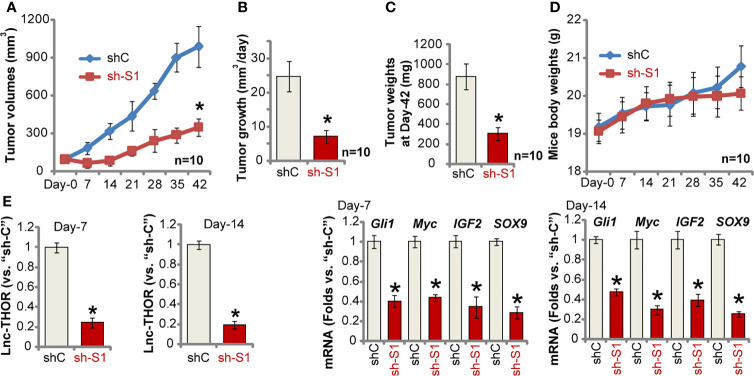
Lnc-THOR shRNA inhibits A549 xenograft tumor growth in nude mice. A549 xenograft-bearing nude mice were subject to intratumoral injection of Lnc-THOR shRNA (“sh-S1”) AAV or the scramble control shRNA (“shC”) AAV. The virus was injected daily for seven consecutive days; Tumor volumes **(A)** and mice body weights **(D)** were recorded every seven days for six rounds (total 42 days, “Day-0” to “Day-42”); The estimated daily tumor growth (in mm^3^ per day) was calculated **(B)**. At Day-42 mice were sacrificed and A549 xenografts were isolated and weighed **(C)**. At experimental Day-7 and Day-14, one tumor of each group was isolated, expression of Lnc-THOR and listed mRNAs **(E)** was tested by qRT-PCR assays. Data were presented as mean ± standard deviation (SD). *P < 0.05 *vs*. “shC” group.

We tested the potential effect of Lnc-THOR on NSCLC cell growth *in vivo*. A549 cells were *s.c.* injected to the flanks of the nude mice. When the tumor volume was close to 100 mm^3^ (“Day-0”), mice were randomly assigned into two groups (with 10 mice per group). The xenograft-bearing mice were subject to intratumoral injection of Lnc-THOR shRNA (“sh-S1”) AAV or the scramble control shRNA (“shC”) AAV. The virus was injected daily for seven consecutive days. As shown, A549 xenografts with Lnc-THOR shRNA AAV injection grew significantly slower than control tumors with shC AAV injection (**Figure 5A**). By using the formula: (Volume at Day-42 subtracting Volume at Day-0)/42, the estimated daily tumor growth (in mm^3^ per day) was calculated. The results further confirmed that Lnc-THOR shRNA virus injection potently inhibited A549 xenograft growth in mice (**Figure 5B**). At Day-42 mice were sacrificed by cervical dislocation, and palpable A549 xenografts were isolated and weighed. Results showed that A549 xenografts with Lnc-THOR shRNA injection were significantly lighter than shC A549 xenografts (**Figure 5C**). The body weights were however not significantly different between two groups (**Figure 5D**).

At experimental Day-7 and Day-14, one tumor of each group was isolated to obtain the tumor tissue lysates. As shown Lnc-THOR was depleted in Lnc-THOR shRNA-injected tumors (**Figure 5E**). In line with the *in vitro* findings, IGB2BP1 target mRNAs, including *Gli1*, *Myc*, *IGF2* and *SOX9*, were decreased as well in Lnc-THOR-silenced A549 xenograft tissues (**Figure 5E**). Collectively, these results show that Lnc-THOR shRNA inhibited A549 xenograft tumor growth in mice.

### Increased Lnc-THOR Expression in NSCLC

**Figure 6:**
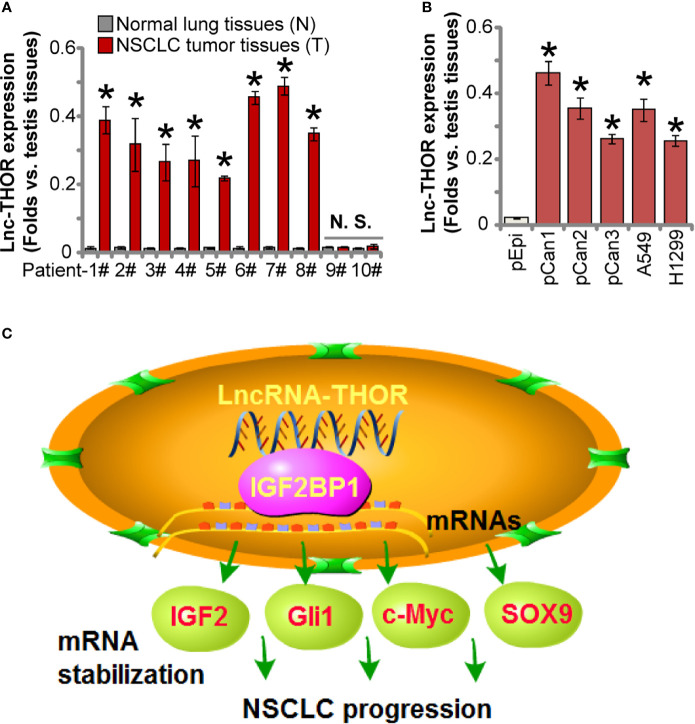
Increased Lnc-THOR expression in NSCLC. RNA was extracted from a total of 10 pairs human NSCLC tumor tissues (“T”) and surrounding normal lung tissues (“N”) **(A)**, the established/primary human NSCLC cells **(B)**, and primary human lung epithelial cells [“pEpi”, **(B)**], Lnc-THOR expression was tested by qRT-PCR assays, with its expression normalized to that of human testis tissues **(A, B)**. The proposed signaling cartoon of this study **(C)**. Data were presented as mean ± standard deviation (SD, n=5). *P < 0.05 *vs*. “N” tissues or “pEpi” cells. “N. S.” stands for non-statistical difference **(A)**. The experiments were repeated three times, with similar results obtained.

At last we tested expression of Lnc-THOR in NSCLC. By employing qRT-PCR assays, we show that Lnc-THOR expression is detected in eight out of 10 human NSCLC tissues (“T”, **Figure 6A**). Lnc-THOR expression in NSCLC tumor tissues was normalized to that in human testis tissues (**Figure 6A**). It was almost not expressed in all cancer-surrounding normal lung tissues (“N”, **Figure 6A**). Lnc-THOR expression was also detected in primary NSCLC cells (“pCan-1/pCan-2/pCan-3”) as well as in established cell lines (A549 and H1299) (**Figure 6B**), but low in primary lung epithelial cells (“pEpi”, **Figure 6B**). Together, Lnc-THOR is overexpressed in NSCLC tissues and cells.

## Discussion

IGF2BP1 is a primary member of IGF2BP RNA-binding family proteins (21, 39), and it regulates stabilization and transcription of several key pro-cancerous/oncogenic genes (21–23, 39). Zhang et al., have shown that IGF2BP1 is important for NSCLC cell progression (40). In NSCLC cells, IGF2BP1 silencing potently suppressed cancer cell proliferation, migration and invasion, as well as induced cell cycle arrest and apoptosis (40). Gong et al., showed that microRNAs-491-5p silenced IGF2BP1 to suppress A549 cell proliferation and migration (41). Studies have demonstrated that the direct binding between Lnc-THOR and IGF2BP1 is critical for IGF2BP1 to maintain its functions (25).

Lnc-THOR was firstly identified in 2017 as a conserved cancer and testis specific Lnc-RNA (24). Since then, the cancer-promoting activity of this LncRNA has been confirmed in multiple malignancies (24, 25, 27–29, 31). Chen et al., reported that Lnc-THOR expression in osteosarcoma (OS) is required for cancer cell growth *in vitro* and *in vivo* (25). In addition, Lnc-THOR is expressed in renal cell carcinoma (RCC). Contrarily, Lnc-THOR silencing or KO suppressed RCC cell proliferation (27). Song et al., have shown that Lnc-THOR increased the stemness of gastric cancer cells by enhancing *SOX9* mRNA stability (28). By promoting c-myc mRNA-IGF2BP1 protein association, Lnc-THOR increased c-myc expression and retinoblastoma cell progression (29). Wang et al., have shown that triptonide inhibited nasopharyngeal carcinoma cell growth by downregulating Lnc-THOR (26). Xue et al., reported that Lnc-THOR is expressed in human glioma, and silencing Lnc-THOR largely inhibited glioma cell survival *via* activating MAGEA6-AMPK signaling (34).

In the present study, we found that Lnc-THOR is overexpressed in NSCLC tissues and cells In established and primary NSCLC cells, Lnc-THOR shRNA or complete KO potently inhibited cell viability, proliferation migration and invasion. Moreover, significant apoptosis was detected in Lnc-THOR-silenced/-KO NSCLC cells. We found that ssDNA contents were significantly increased in Lnc-THOR shRNA or KO cells, which could initiate a DNA damage response to provoke apoptosis. Therefore, ssDNA formation could be an important cause of Lnc-THOR depletion-induced apoptosis induction in NSCLC cells. The underlying mechanisms warrant further characterizations. Conversely, forced Lnc-THOR overexpression, by a lentiviral construct, accelerated NSCLC cell proliferation, migration and invasion. *In vivo*, Lnc-THOR shRNA potently inhibited A549 xenograft tumor growth the nude mice. These results suggested that Lnc-THOR could be an important therapeutic target and a promising diagnosis marker for NSCLC.

Here we provided evidence to support that Lnc-THOR-driven NSCLC cell growth is through binding to IGF2BP1 (see the proposed signaling cartoon in **Figure 6C**). RNA-IP and RNA pull-down results showed a direct binding between Lnc-THOR and IGF2BP1 protein in NSCLC cells. mRNA expression of IGF2BP1 target mRNAs, including *IGF2*, *Gli1*, *Myc* and *SOX9*, were significantly downregulated in Lnc-THOR-silenced/-KO NSCLC cells, but increased after Lnc-THOR overexpression. IGB2BP1 target mRNAs were robustly decreased in Lnc-THOR-silenced A549 xenograft tissues. Mimicking Lnc-THOR depletion-induced anti-cancer activity, CRISPR/Cas9-induced IGF2BP1 KO potently inhibited NSCLC cell proliferation and migration. More importantly, Lnc-THOR silencing and overexpression were ineffective in IGF2BP1-KO NSCLC cells. Moreover, forced IGF2BP1 overexpression failed to rescue proliferation, migration and invasion of Lnc-THOR-KO NSCLC cells. Therefore, by direct associating with IGF2BP1 Lnc-THOR promotes NSCLC cell growth.

## Conclusion

These results highlighted the ability of Lnc-THOR in promoting NSCLC progression by associating with IGF2BP1, suggesting that Lnc-THOR represents a promising and novel therapeutic target of NSCLC.

## Data Availability Statement

The original contributions presented in the study are included in the article/**Supplementary Material**. Further inquiries can be directed to the corresponding authors.

## Ethics Statement

The studies involving human participants were reviewed and approved by the Ethics Committee of Zhengzhou University. The patients/participants provided their written informed consent to participate in this study. All animal studies were performed according to the standards of IACUC of Zhengzhou University, with the protocols approved by the Ethics Committee of Zhengzhou University.

## Author Contributions

All the listed authors in the study carried out the experiments, participated in the design of the study and performed the statistical analysis, conceived of the study, and helped to draft the manuscript. All authors contributed to the article and approved the submitted version.

## Funding

This work is supported by the Key R & D and promotion projects in Henan Province (212102310192) and Henan Medical Science and technology research plan (LHGJ20190218). The funders had no role in study design, data collection and analysis, decision to publish, or preparation of the manuscript.

## Conflict of Interest

The authors declare that the research was conducted in the absence of any commercial or financial relationships that could be construed as a potential conflict of interest.

## Publisher’s Note

All claims expressed in this article are solely those of the authors and do not necessarily represent those of their affiliated organizations, or those of the publisher, the editors and the reviewers. Any product that may be evaluated in this article, or claim that may be made by its manufacturer, is not guaranteed or endorsed by the publisher.
