# Supplementary figures and images for: Long Non-Coding RNA THOR Depletion Inhibits Human Non-Small Cell Lung Cancer Cell Growth
Source: Front Oncol. 2021 Nov 17;11:756148. doi: 10.3389/fonc.2021.756148 (PMC8635526; doi:10.3389/fonc.2021.756148)

Figure 2B

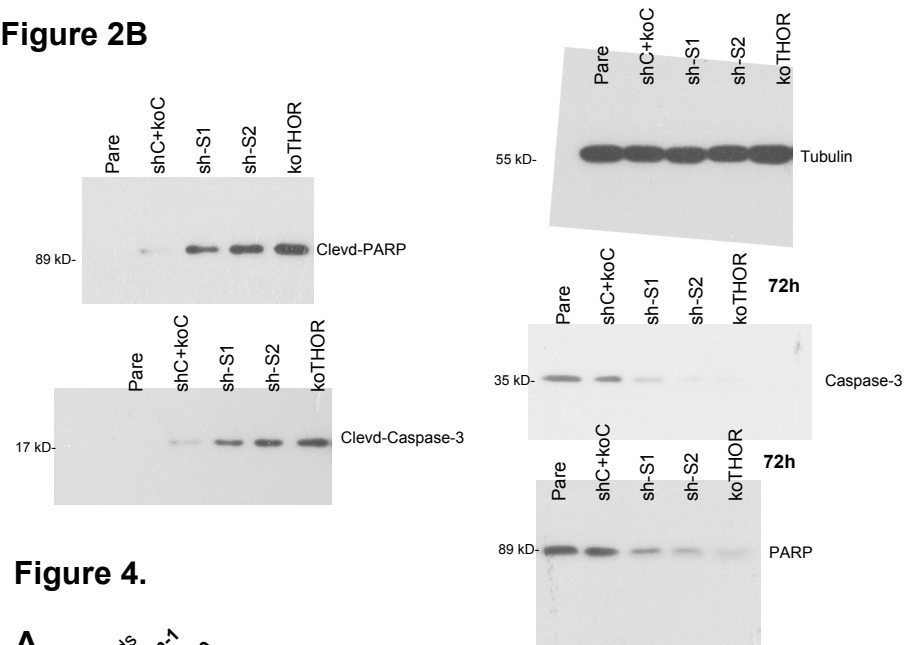

Figure 4.

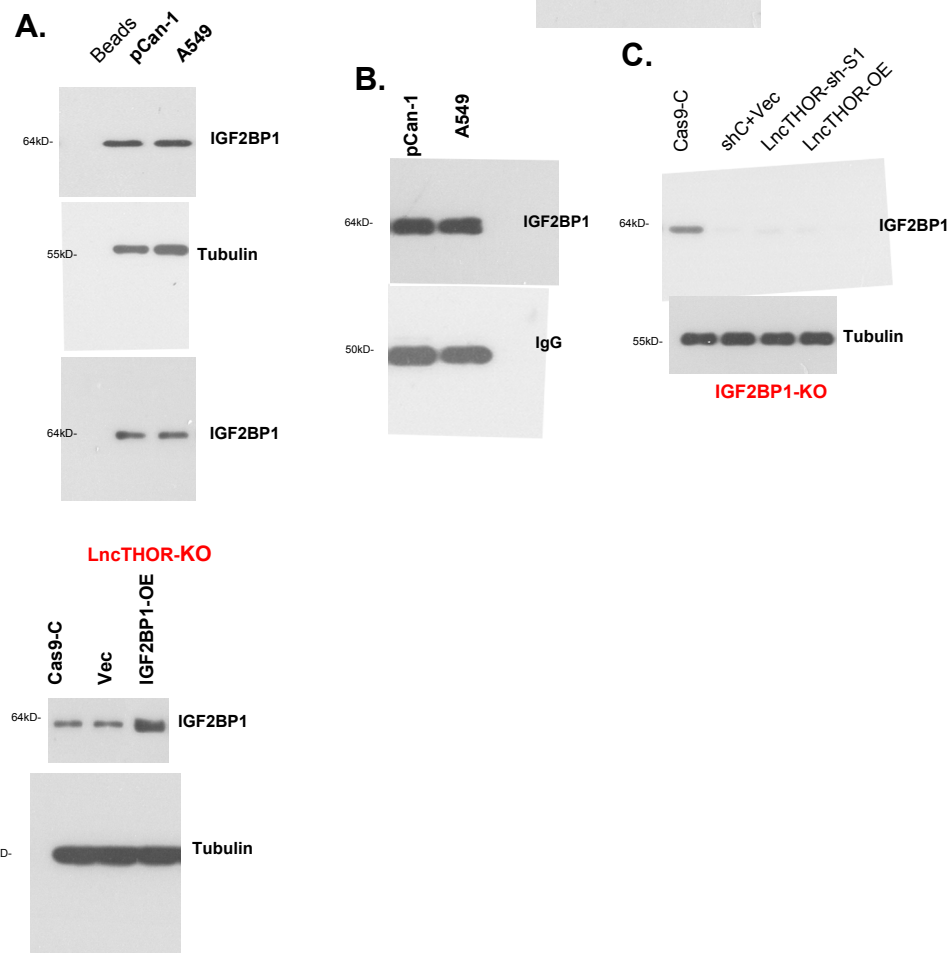

Supplement: Supplementary file 1 [file DataSheet_1.pdf]
